# Pre‑operative mean platelet volume is associated with overall survival in patients with IDH‑wildtype glioblastoma undergoing maximal safe resection

**DOI:** 10.3892/ol.2024.14709

**Published:** 2024-09-30

**Authors:** Silvia Snider, Pierfrancesco De Domenico, Francesca Roncelli, Andrea Bisoglio, Matteo Braga, Anna Ghelfi, Lina Raffaella Barzaghi, Cinzia Mura, Pietro Mortini, Filippo Gagliardi

**Affiliations:** 1 Department of Neurosurgery and Gamma Knife Radiosurgery, San Raffaele Scientific Institute, Istituto di Ricovero e Cura a Carattere Scientifico (IRCCS), I-20132 Milan, Italy; 2 Department of Neurosurgery, Duke University, Durham, NC 27701, USA

**Keywords:** glioblastoma, platelets, mean platelet volume, high-grade glioma, activated platelets

## Abstract

Glioblastoma (GBM) is the most common, fast-growing, and aggressive malignant primary CNS tumor, with a survival time of ~15 months despite the use of surgery and adjuvant treatments. In recent years, there has been a growing interest in exploring the potential contribution of hemostasis and platelet activation in GBM biology. The present study assessed the association between the pre-operative coagulation profile [as indicated by prothrombin time (PT) ratio and activated partial thromboplastin time (aPTT) ratio], overall platelets (PLT) count and the mean platelet volume (MPV) with tumoral characteristics and overall survival in patients with isocitrate dehydrogenase-wildtype (IDH-wt) GBM.

A total of 167 adult patients undergoing maximal safe resection of newly diagnosed World Health Organization grade 4 IDH-wt glioblastoma were included. The variables of interest (MPV, PT ratio, and aPTT ratio) were dichotomized at the median, while the overall PLT count was split using the central distribution (10th to 90th percentile). Correlation analyses of markers with tumoral and demographic characteristics, Kaplan Meier survival analysis, and Cox multivariate regression analysis were conducted to assess the single contributions of these parameters in building a predictive model of overall survival (OS) in these patients.

The mean baseline MPV correlated with increasing age (r=0.18, P=0.01), the overall fluid-attenuated inversion recovery tumoral volume (r=0.17, P=0.02), and lesion T1-weighted post-contrast sequence (T1-CE) volume (r=0.19, P=0.01). The median OS in the whole cohort of patients with GBM was 14.4 months (95% CI 12.9-17.6). Patients with MPV >10.3×10^−15^ l had a median OS of 13.4 months (95% CI 10.6-17.6) compared with 14.5 months (95% CI 13.4-20.6) in patients with MPV ≤10.3×10^−15^ l (P=0.028). Similarly, shorter OS was recorded in patients with PT ratio >1.01 (12.3 months, 95% CI 10.2-15.1 vs. 17.6 months, 95% CI 13.4-20.6; P=0.006) and PLT count out-of-range 165–300×10^9^/l (11.5 months, 95% CI 8.8-16.3 vs. 14.7 months, 95% CI 13.4-19.1; P=0.026). A subgroup analysis of patients >65 years of age confirmed baseline MPV >10.3 10^−15^ l was associated with shorter OS (9.4 months, 95% CI 8.1-13.4) compared with 13.3 months (95% CI 11.3-32.3, P=0.028) for those with MPV ≤10.3×10^−15^ l. Baseline-increased MPV showed an independent predictive role for poor survival (HR, 1.56; 95% CI 1.13-2.16; P=0.006) in multivariate analysis accounting for age, gender, performance status, extent or resection, adjuvant therapies, and tumoral molecular and radiological characteristics, whereas PLT count within the central range predicted longer OS (HR, 0.26; 95% CI 0.13-0.54; P<0.001).

The present study indicates a possible association between tumoral burden and systemic hemostasis activation in patients with IDH-wt GBM. Increased MPV and deranged PLT outside the central range demonstrated an independent role in predicting shorter OS, which was even more prominent among older patients. These findings require additional studies to further validate these results and specifically characterize GBM pathological features of aggressiveness related to hemostasis activation, neo-angiogenesis, the tumor immune microenvironment, and their effect on response to treatments and OS.

## Introduction

Glioblastoma (GBM) is the most common, fast-growing, and aggressive malignant primary CNS tumor worldwide, accounting for 48.6% of malignant central nervous system tumors (1), with an incidence of 3–4 cases per 100,000 person-years (2). The median survival time is ~15 months, despite the use of surgery and adjuvant treatments (3). The diagnosis of GBM is currently defined by the 2021 World Health Organization (WHO) classification of CNS tumors (4), which uses integrated molecular marker analysis and chromosomal aberrations. GBM WHO grade 4 is defined as a diffuse astrocytic glioma, with isocitrate dehydrogenase (IDH), histone 3 (H3) and wild type (wt) features, and characterized by prominent cellular and nuclear atypia, frequent mitotic activity, necrosis, and vascular proliferation. Telomerase reverse transcriptase (TERT) promoter mutation, epidermal growth factor receptor (EGFR) gene amplification, and +7/-10 chromosome copy-number changes characterize the specific molecular features. Current GBM treatment is multimodal and has not been substantially changed since 2005, despite notable efforts in neuro-oncological research. GBM treatment consists of maximal safe resection surgery, followed by concomitant adjuvant radiotherapy and chemotherapy (3).

In recent years, there has been a growing study of the potential contribution of hemostasis and platelet activation in cancer biology (5–18). Although the exact interplay between circulating blood cells, peritumoral immune infiltrate, and intra-tumoral microenvironment is still not fully understood, the relevance of cancer-induced systemic activation of soluble and cellular hemostasis components in promoting tumor growth and progression has been increasingly emphasized (19). The mechanism of platelet-induced tumorigenesis and progression has only been partly elucidated and is thought to be mainly associated with the role served by activated platelets in sustaining tumoral neo-angiogenesis via the release of pro-angiogenic factors contained in α-granules. Additionally, the excessive and unbalanced release of pro-thrombotic molecules and pro-inflammatory cytokines results in changes in the thrombotic/fibrinolytic balance, and the recruitment of circulating leukocytes, contributing to their extravasation and polarization towards immune-permissive subpopulations which contribute to immune escape (20,21). These mechanisms, together, contribute to the promotion of cellular evasion and metastatic seeding (19).

Platelet number and morphology are evaluated through routine, low-cost blood tests, such as platelet count (PLT count) and mean platelet volume (MPV). The latter is considered a feature of platelets' activation, with increased MPV indicating activation of a large number of platelets (22). Given this, the PLT count and MPV have been used as diagnostic markers in solid tumors, as they have proved helpful in distinguishing malignant from benign lesions in hepatic, nasopharyngeal, and colorectal cancer (12,14). Additionally, MPV has been reported to show prognostic value in predicting shorter OS in certain types of solid cancers including esophageal, gastric (23), pancreatic (24), lung (25), breast, colorectal, head and neck, hepatic, urothelial cancer, melanoma and osteosarcoma, and hematologic cancers including multiple myeloma and diffuse large B-cell lymphoma (23–25).

The present study addressed the association of baseline pre-operative PLT count, MPV, coagulation profile including prothrombin time (PT), prothrombin ratio (PT ratio), activated partial thromboplastin time (aPTT), and aPPT ratio with demographic and tumoral parameters, and their impact on OS in patients with GBM.

## Materials and methods

### Study design, patient selection, and data retrieval

The present study was a single-center, retrospective, non-controlled clinical study, designed to assess the role of platelet activation and the coagulation profile in patients with GBM. Patients with adequate clinical follow-up, who underwent maximal safe resection of newly diagnosed grade 4 isocitrate dehydrogenase-wildtype (IDH-wt) glioblastoma at IRCCS San Raffaele Hospital (Milan, Italy) between 2016 and 2023 were included. Pediatric patients (<18 years of age) and patients who demonstrated unresectable disease, underwent biopsy only, had IDH-mutant tumors, or recurrent GBM were excluded from the current analysis.

Diagnoses were originally performed according to the 2016 World Health Organization (WHO) classification of tumors of the central nervous system (26) or the 2021 WHO edition (4), depending on when surgery was performed. As the intent of the present study was to analyze a homogenous population of ‘primary’ (i.e., IDH-wt) GBM, cases defined as ‘secondary’ or ‘IDH-mutated’ lesions were excluded (27). Between January 2016 and December 2021, only patients who presented a diagnosis of IDH-wt GBM through immunohistochemical detection of the absence of IDH1 R132 mutations were enrolled (n=136).

After January 2021, only GBM IDH-wt patients according to the 2021 WHO classification were included (n=31). IDH1-2 mutational status was determined, and all cases whose IDH status was not available were excluded. Pathological and molecular findings such as O-6-methylguanine-DNA-methyltransferase (MGMT) promoter methylation status, Ki-67 index, and p53 expression were reported. Testing for TERT mutation was not routinely performed before 2021 at the IRCCS San Raffaele Hospital, therefore only a subset of patients in this cohort had TERT mutation status data. Specimens were processed and analyzed at the Department of Pathology (San Raffaele Scientific Institute) as per standard of care methods for diagnosis, according to WHO standards (4,26). Briefly, for immunohistochemistry, 2 µm thick paraffin-embedded representative tissue sections were de-waxed in xylene and rehydrated using 3×10 min 99% ethanol and 2×10 min 96% ethanol washes. Endogenous peroxidase activity was blocked with 0.3% H_2_O_2_ in methanol for 20 min. Antigen retrieval (when necessary) was performed by using a microwave oven or a thermostatic bath at 98°C for 40 min in either 1.0 mM EDTA buffer (pH 8.0) or 1 mM Citrate buffer (pH 6.0). Sections were then washed in TBS (pH 7.4), and incubated in the specific primary antibody at 37°C for 30 min. The signal was revealed using the DAKO Envision + System-HRP Labelled Polymer Anti-Rabbit or Anti-Mouse (Novocastra™) followed by DAB as chromogen and hematoxylin as counterstain, according to the manufacturer's instructions. The primary antibodies used were as follows: mouse monoclonal anti-p53 (Prediluted; clone DO-7; cat. no. 800-2912; Roche Tissue Diagnostics; Roche Diagnostics, Ltd.), rabbit monoclonal anti-Ki-67 (clone 30-9; Prediluted; cat. no. 790-4286; Roche Tissue Diagnostics; Roche Diagnostics, Ltd.), rabbit polyclonal anti-ATRX (1:300; cat. no. PA5-21348; Sigma-Aldrich), rabbit monoclonal anti-GFAP (Prediluted; clone EP672Y; cat. no. 760-4345; Roche Tissue Diagnostics; Roche Diagnostics, Ltd.), mouse monoclonal anti-IDH1 R132H (1:100; clone H09; cat. no. DIA-H09; Dianova GmbH). In cases with negative immunostaining for IDH1-R132H, IDH1/2 mutational status was assessed using Illumina MiSeq (Myriapod NGS Kit Cancer panel DNA, Diatech Pharmacogenetics) according to the manufacturer's protocol. Evaluation of O6-methylguanine DNA methyltransferase (MGMT) promoter methylation status was performed using a pyrosequencing methylation assay using the MGMT PLUS kit CE IVD (Diatech Pharmacogenetics) according to manufacturer's instructions.

Tumoral volumes were calculated on preoperative MRI imaging using Cranial Planning Anatomical Mapping (version 1.1.1.8) and SmartBrush (version 3.0.0.92) (Brainlab AG) to assess fluid-attenuated inversion recovery (FLAIR) and T1-weighted post-contrast sequences (T1-CE). The presence of satellite lesions was defined as the occurrence of hyperintense signals in the FLAIR sequence non-contiguous with the target lesion and therefore outside the planned surgical field. The extent of resection (EOR) was calculated on MRI imaging performed within 72 h post-surgery when available, or on MRI scans performed for RT planning, before any additional treatment as per Response Assessment in Neuro-Oncology guidelines (28). Baseline and follow-up clinical data were retrospectively retrieved from clinical records and included age, gender, and performance status using the Karnofsky score (29).

### Blood sampling

Preoperative peripheral blood samplings including PLT count (normal range, 130–400×10^9^/l), MPV (normal range 9.1-12.5×10^−15^ l), PT ratio (normal range 0.85-1.18) and aPTT ratio (normal range 0.8-1.23) were routinely performed upon hospital admission, before any treatment, and within the 24 h period preceding surgery. Specimens were processed immediately after collection in the IRCCS Ospedale San Raffaele Hospital central analysis laboratory as per the normal standard of care.

### Statistical analysis

Statistical analysis was performed using R Core Team (2022) (30), using survival (version 3.5-5) (31), ggsurvfit (version 1.0) (32), corrplot (version 0.92) (33), and ggplot2 (version 1.0) (34) packages. Categorical variables are reported as absolute numbers and percentages whereas continuous variables are reported as mean and standard deviation or median and interquartile range. The difference in baseline characteristics and the unadjusted univariate analyses were performed using Student's t-test or Mann-Whitney U test in accordance with the normality of the distribution. χ^2^ and Fisher's exact test, were used depending on the expected count.

Pearson's correlation test was used to infer associations between demographics, clinical and serum markers variables, and mortality. The continuous variables of interest (MPV, PT, and aPTT) were dichotomized at the median, whereas the PLT count was taken from the central range (10th to 90th percentile) and two-sided lower and upper ‘out-of-range’ tails. The Kaplan-Meier method was used to estimate OS in the study population using the newly dichotomized variables. To address the association between serum markers and age, a subgroup analysis of an older patient population (>65 years) was conducted utilizing the same cut-offs for the continuous variables. The log-rank test was used to analyze differences between groups. Univariate and multivariate Cox regression analyses were used to detect variables associated with increased overall survival. P<0.05 was considered to indicate a statistically significant difference. A two-stage procedure for comparing hazard rate functions was applied using the TSHRC (version 0.1-6) package (35), when the proportional hazard assumption was violated.

## Results

### Patients, pathological characteristics, and treatments

A total of 167 patients with WHO grade 4 IDH-wt GBM were included in the present study. The mean age of the patients was 63±10.5 years. Most of the patients were male (n=111, 66%) and aged <65 years old (n=92, 55.1%). Overall, patients displayed a good functional status using Karnofsky performance status (KPS >80) in 63.4% of cases. Patients were followed up clinically for a median period of 12.8 months. A comprehensive summary of the baseline characteristics of included patients, tumors, and peripheral markers is presented (Table I). Assessment of MGMT promoter status was available in 134 patients (80%) and revealed promoter hypermethylation in 49/134 (37%) patients. The quantitative analysis of ki67 and p53 reported a mean of 31±18 and 20±24% immunoreactive cells, respectively. EGFR amplification and TERT mutation data were available in <10% of patients and therefore were not included in the present analysis.

The EOR was retrieved for all included patients and calculated as complete in 69 (41%), near total in 51 (31%), partial in 13 (8%), and subtotal in 34 (20%). Post-operative treatment data were available for 149 patients (89%). Among them, the post-operative concurrent radiotherapy (RT) and chemotherapy with temozolomide (TMZ) regimen were completed in 115 (77%) patients, conversely, the remaining 34 (23%) only received RT or did not complete the concurrent RT/TMZ regimen for TMZ. Data on additional adjuvant therapies was available for 129 patients (73%). Most of these received adjuvant TMZ (n=107, 83%) completing 6 cycles in 42% of cases and 12 cycles in 7% of cases.

### Cut-off selection and baseline characteristics of pre-operative laboratory parameters

The blood marker values of MPV, PT, PT ratio, aPTT, and aPTT ratio were split at the median. The resultant thresholds for survival analysis were as follows: MPV, 10.3×10^−15^ l; PT, 13.2 sec; PT ratio, 1.01; aPTT, 28.4 sec; and aPTT ratio, 0.94. The PLT count was split up using the central distribution (10th to 90th percentile) which corresponded to the range of 165–300×10^9^/l.

The baseline demographic characteristics of patients were not significantly different between patients with high and low MPVs, except for age (64.7±10.8 vs. 60.8±9.96 years, respectively; P=0.01). No significant differences in baseline performance status, steroid use (dexamethasone), inflammatory markers (white blood cell count and neutrophil count), or molecular signature were detected. Patients with MPV >10.3×10^−15^ l had slightly larger T1-CE volumes (P=0.07). Univariate analysis for MPV is presented (Table II).

Similarly, patients with increased PT ratio showed slightly greater mean age (64.8±10.4 vs. 60.4±10.2 years, P=0.009) compared with patients with lower values. All other parameters were otherwise comparable in these patients as well as in the other study groups (aPTT ratio and PLT count).

### Survival analysis

#### Pre-operative laboratory parameters

**Figure 1.**
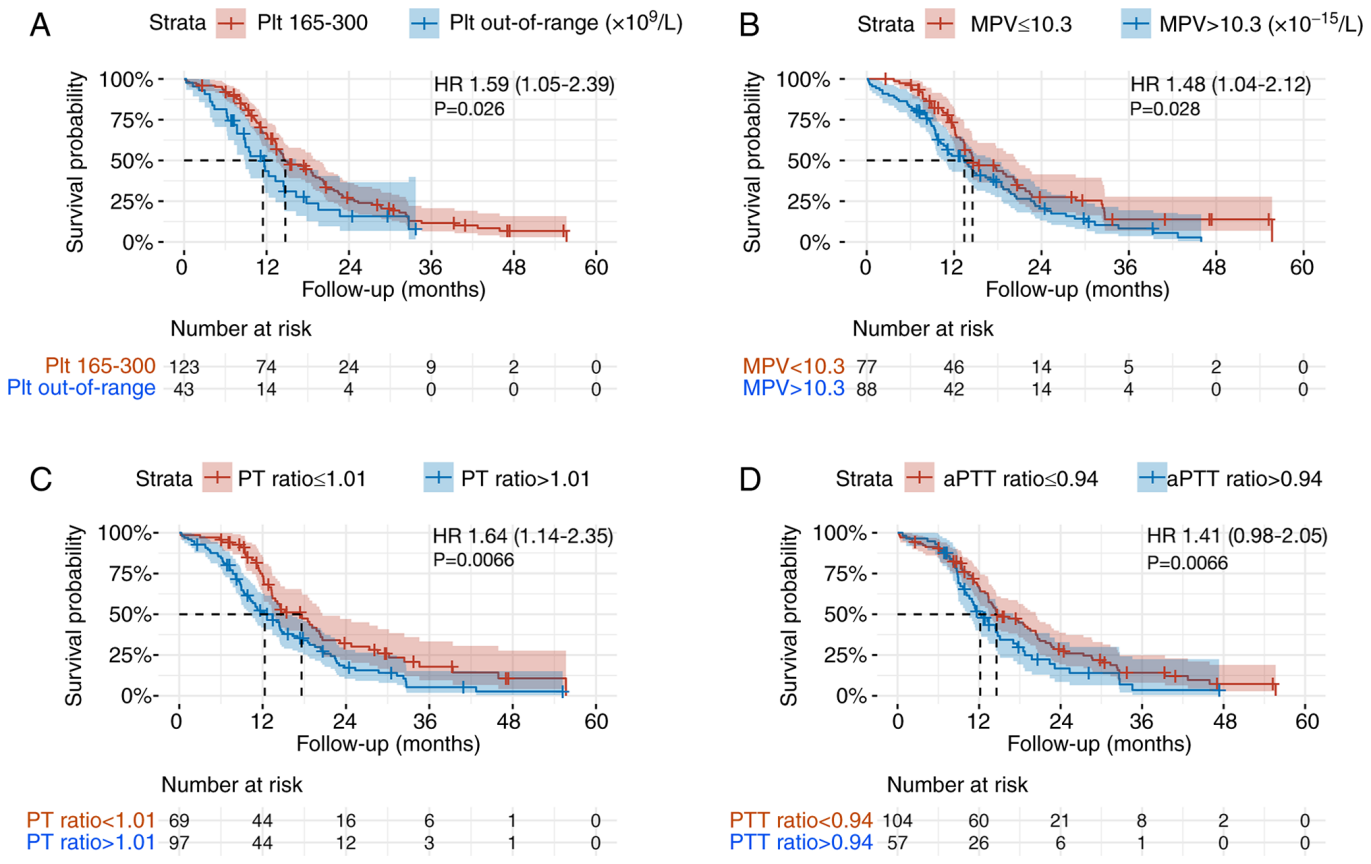
Kaplan-Meier curves for overall survival analysis. (A) PLT count normal range (165–300×10^9^/l) vs. out-of-range. (B) MPV high vs. low. (C) PT-ratio high vs. low. (D) aPTT-ratio high vs. low. P-values represent log-rank test results. HR, hazard ratio with 95% CI interval; PLT, platelet; MPV, mean platelet volume; aPTT, activated partial thromboplastin time.

The median OS in the whole cohort of patients with GBM was 14.4 months (95% CI, 12.9-17.6). The median OS of patients with a PLT count out of the central range was significantly shorter than that of patients with PLT in the central range (165–300 10^9^/l) with a median OS of 11.5 months (95% CI, 8.8-16.3) compared with 14.7 months (95% CI, 13.4-19.1) (P=0.026, Fig. 1A).

Patients with MPV >10.3×10^−15^ l had a median OS of 13.4 months (95% CI, 10.6-17.6) compared with 14.5 months (95% CI, 13.4-20.6) in patients with MPV ≤10.3×10^−15^ l (P=0.028, Fig. 1B). Similarly, patients with PT ratio >1.01 achieved a median OS of 12.3 months (95% CI, 10.2-15.1) compared with 17.6 months (95% CI, 13.4-20.6) in patients with PT ratio ≤1.01 (P=0.006, Fig. 1C). However, the difference in median OS of patients with baseline aPTT ratio >0.94 of 12.2 months (95% CI: 10.2-17) was not significantly different to the 14.6 months (95% CI, 13.4-20.4) of patients with aPTT ≤0.94 (P=0.06, Fig. 1D).

### Age and performance status

Patients aged >65 years had a median OS of 11 months (95% CI, 9.5-14.7) which was significantly shorter when compared with the 20.2 months (95% CI, 14.7-23) in younger patients (P<0.001; Fig. S1A).

The median OS for patients with low (<70) and high (≥80) KPS were 12 (95% CI, 9.6-16.3) and 14.7 months (95% CI, 13.4-19.8), respectively (P=0.03; Fig. S1B).

### Gender, MGMT status, and satellite lesions

Survival analysis for gender (P=0.57, Fig. S1C) and MGMT methylation (P=0.14, Fig. S1D) status did not indicate any significant differences in OS in this patient cohort. However, female patients did achieve a gain in OS against males in the subgroup of patients with MGMT promoter methylation (OS 24.8 months with 95% CI 14.6-32 vs. 14.5 months with 95% CI 13.3-20.2; P=0.04; Fig. S1E). The presence of satellite FLAIR lesions was associated with a significantly shorter OS, of 11.5 months (95% CI, 8.7-14.4) for the additional areas group compared with 15.1 months (95% CI, 13.4-18.7) for the unifocal group (P=0.02; Fig. S1F).

### Subgroup analysis in patients aged >65 years

Additional survival analysis in the older subgroup of patients (>65 years of age) was conducted to evaluate the association between increased age and baseline MPV and PT. Besides a worse overall performance status (KPS) compared with younger patients (P=0.001), no other variables including tumoral volumes, mutations, ki67 status, or demographic parameters differed in the two populations.

PLT count within range and aPTT ratio did not show any significant difference in terms of OS (Fig. S2A and D). However, patients with MPV >10.3×10^−15^ l demonstrated a shorter median OS of 9.4 months (95% CI, 8.1-13.4) compared with 13.3 months (95% CI, 11.3-32.3) for patients with MPV ≤10.3×10^−15^ l (P=0.028, Fig. S2B). Similarly, patients with a high PT ratio (>1.01) achieved OS of 8.9 months (95% CI, 8.1-11.3) compared with 13.8 months (95% CI, 11.4-23.1) for patients with a low PT ratio (P=0.01, Fig. S2C).

Additionally, to further test the interaction of age and MPV in OS, the relative mortality rate of patients with increased or decreased MPV values in the 2 subgroups of patients with different ages, were calculated. Within the elderly group (>65 years) the mortality rate for patients with increased vs. decreased MPV was 31/32 (97%) vs. 11/15 (73%). In the younger group of patients (<65 years) it was 44/58 (76%) vs. 43/62 (69%) and the effect of age and MPV on OS rate was not statistically significant (P=0.092).

### Correlation and regression analyses

**Figure 2.**
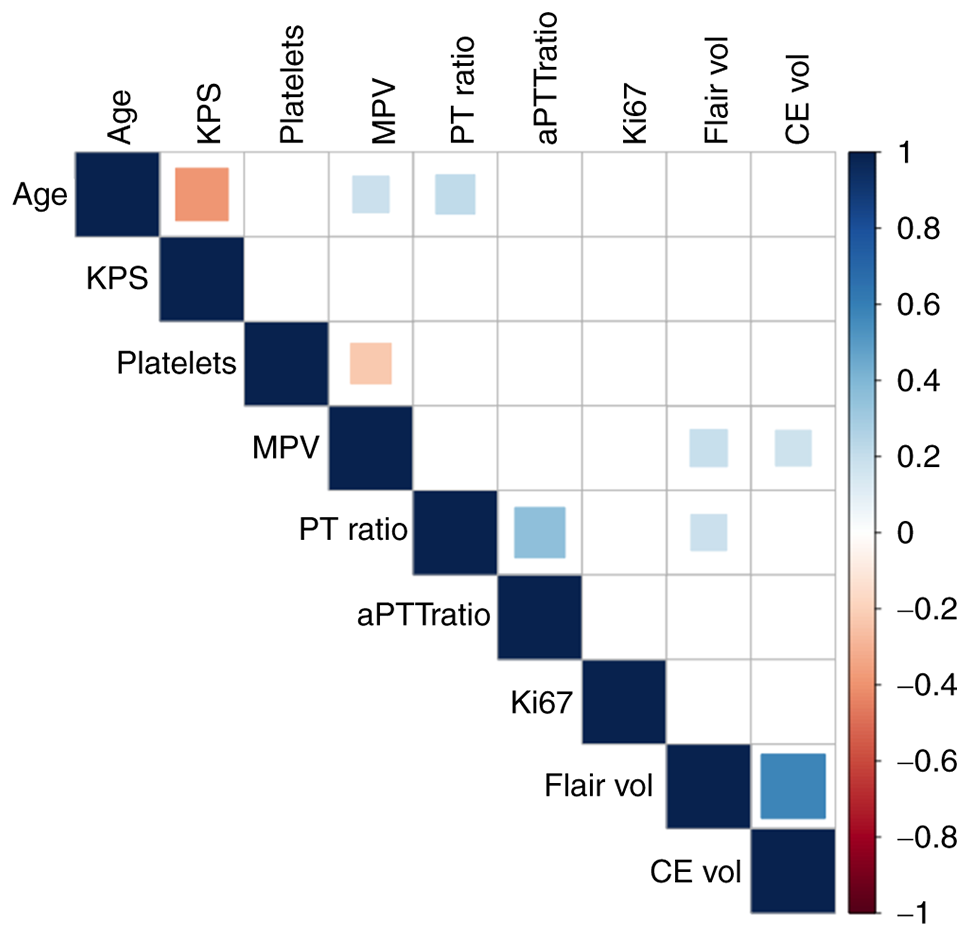
Correlogram presenting results from Pearson correlation analysis, blank squares represent non-significant associations (P>0.05), red squares represent significant (P<0.05) negative associations, whereas blue squares represent significant (P<0.05) positive associations.

Significant associations were observed between increasing age and lower KPS (r=−0.38, P<0.001), increased MPV (r=0.18, P=0.01), and increased PT ratio (r=0.21, P=0.005). Additionally, MPV values were significantly associated with the FLAIR (r=0.17, P=0.02) and the T1-CE volumes (r=0.19, P=0.01). A similar association was observed between the PT ratio and FLAIR volume (r=0.18, P=0.02). The MPV was also significantly inversely correlated with PLT (r=−0.22, P=0.003). Correlation analyses between demographic characteristics, baseline platelet and coagulation parameters, and tumoral burden were summarized (Fig. 2).

In terms of post-operative treatments, no significant associations between MPV values or PLT count and completion rate of concurrent RT/chemotherapy schedules were demonstrated (OR 0.84, 95% CI 0.56-1.25, P=0.39; and OR 0.99, 95% CI 0.99-1.00, P=0.77; respectively). Similarly, the completion of at least 6 cycles of adjuvant temozolomide (TMZ) was not associated with baseline MPV (OR 0.88; 95% CI, 0.59-1.32; P=0.55) or PLT count (OR 1.00; 95% CI, 0.99-1.01; P=0.08). Moreover, no significant association was observed between the number of adjuvant TMZ cycles and pre-operative MPV (r=−0.27, P=0.41; OR 0.94, 95% CI 0.61-1.45, P=0.80) or PLT count (r=−0.001, P=0.81; OR 1.00, 95% CI 0.99-1.00, P=0.73).

The mortality rate among patients with higher MPV values was significantly different from that of patients with lower MPV values (58.1 vs. 41.86%; OR 2.12, 95% CI 1.01-4.45; P=0.04).

### Survival Cox regression analysis

Cox regression between preoperative platelet parameters and adjuvant chemotherapeutic regimen indicated that all included parameters retained a significant value in predicting OS. Specifically, increased MPV had a detrimental effect (HR 1.49; 95% CI, 1.21-1.83; P<0.001), while in-range-PLT (HR 0.35; 95% CI, 0.21-0.58; P<0.001), concurrent RT/TMZ (HR 0.37; 95% CI, 0.22-0.60; P<0.001) and adjuvant TMZ (HR 0.48; 95% CI, 0.29-0.78; P<0.001) predicted better OS (Table III).

In addition, multivariate Cox regression analysis of OS accounting for age, gender, pre-operative performance status, and tumoral characteristics (R^2^=0.70, P<0.001) indicated high MPV as an independent predictive variable for poor OS (HR 1.56; 95% CI, 1.13-2.16; P=0.006) together with increased age (HR 1.03; 95% CI, 1.01-1.06; P=0.01). Platelet counts within the central range were confirmed as predictors of increased OS (HR 0.26; 95% CI, 0.13-0.54; P<0.001), together with complete surgical resection (HR 0.52; 95% CI, 0.30-0.90; P=0.01), and completion of post-operative concurrent RT/TMZ (HR 0.24; 95% CI, 0.13-0.45; P<0.001) (Table IV).

## Discussion

This retrospective study evaluated the association of baseline peripheral markers of hemostasis and platelet activation, and relevant oncological outcomes in patients with GBM. The analysis indicated that higher MPV values were associated with lower OS and a higher mortality rate compared with patients with lower MPV levels. Other parameters that demonstrated a negative association with OS were: PLT count outside the central distribution and increasing age. Other markers including PT and aPTT ratio did not demonstrate a strong predictive role in multivariate analysis, to account for other relevant demographic, clinical, and molecular variables. It can be hypothesized that the higher mortality rate observed among the patients with increased MPV reflected the reduced OS time observed in this cohort. These findings highlight the need for additional studies to investigate the role of circulating hemostasis and platelet mediators in the elucidation of mechanisms of glioblastoma aggressiveness.

## Diagnostic and prognostic role of baseline MPV in other tumors

The concept of platelets being associated with tumorigenesis and tumor progression has been previously reported, with growing reports suggesting a diagnostic and prognostic role for platelet counts and MPV in oncology research (5–16,18). An increased MPV value represents an index of platelet activation and has been previously investigated as a diagnostic marker in solid tumors including breast (11,36–38), endometrial (39–44), gastric (9,45,46), colon (47), esophageal (48,49), and lung cancer (50–52). However, the association between MPV and the presence of cancer has not always been unequivocal, with other studies reporting an opposing relationship with decreased MPV value in patients affected by gastric (53), colon (54), locally advanced esophageal cancer (55), and renal cell carcinoma (6). A wide meta-analysis on the topic conducted in 2016 (56) concluded that the baseline MPV tends to be higher in oncological patients when compared with healthy subjects and that its mean value decreased after treatment, which suggested a proportional tumoral activation of circulating platelets, in at least some of the investigated tumors.

The MPV has also been reported as a prognostic factor for survival in certain types of solid cancers including esophageal (23), gastric, pancreatic (24), lung (25), breast, colorectal, head and neck, hepatic, urothelial cancer, melanoma, osteosarcoma and hematologic malignancies (23–25), again with equivocal results. In a large meta-analysis (23) of 38 studies including 9,894 patients with both solid and hematological tumors, authors reported that pre-treatment MPV value was not broadly associated with OS, with certain reports suggesting a worse prognosis in patients with increased MPV (particularly in gastric and pancreatic cancer), while this effect seemed opposite in lung cancer (25). In a second review that investigated the role of MPV (24), it was reported that most studies on colon carcinoma reported an unfavorable prognostic role for increased MPV. Based on the results of the present study, it was hypothesized that these mixed results could be partially explained by the existence of a different grade of platelet activation as part of the tumorigenesis process, which is peculiar to each tumor, and is the result of the unique metabolic and immune interplay that sustains the cellular growth and invasion. For this reason, markers of platelet activation may not have a universal role in the detection and grading of patients' prognosis in patients with cancer, rather their significance needs to be understood and validated for each unique type of tumor.

## MPV and platelet activation in the pathophysiology of GBM

The role of platelets in the intricate interplay of the peritumoral microenvironment is still largely unknown, however increasing studies have reported their active involvement in promoting inflammation, immunosuppression, and neo-angiogenesis in patients with GBM. Platelets can be activated by numerous chemical or mechanical signals and can potently interact with circulating leukocytes. Upon activation, platelets express CD40L and P-selectin which directly recruit circulating leukocytes (20,21) and further promote white cell extravasation by inducing the upregulation of endothelial P-selectin, E-selectin, I-CAM1, and V-CAM1 molecules (19). The role of platelets in initiating or promoting the immune response is not limited to leukocyte adhesion and extravasation but is also intimately related to the active recruitment of specific subpopulations through the release of the chemokine (C-X-C motif) ligand (CXCL)1, CXCL4, CXCL5, CXCL7, and CXCL12 chemokines. It has been previously reported that high levels of TGF-β and CD40L have a potent immunosuppressive activity by promoting a decrease in tumor-infiltrating CD8^+^ T cell levels and a relative increase of immunosuppressive CD4^+^FOXP3^+^T-regulatory lymphocytes (57) and M2 macrophages (58). Additionally, it is well established that platelet activation is one of the most potent neo-angiogenesis events, which is mediated by the release of vascular endothelial growth factor VEGF and FGF-2 from α granules of activated platelets, IL-8, IL-10, and prostaglandin E2 (59). This cascade of events has been observed in glioma models (57) showing significant platelet activation, soluble CD40 release, and an increase in immunosuppressive T-reg cells, neo-angiogenesis, vascular damage, and cellular evasion leading to tumor progression. In this context, monitoring MPV and PLT count could be used to estimate the systemic activation of platelets and therefore of tumor-promoting mechanisms being active in sustaining the survival and growth of tumoral cells.

## Prognostic role of platelet activation in GBM

Only a few studies (60–62) have previously specifically addressed the relationship between platelet activation markers and relevant oncological outcomes in GBM. Campanella *et al* (62) reported that tumoral cells promoted the systemic activation of platelets and highlighted the key role of VEGF and sphingolipid signaling pathways in GBM tumorigenesis. More specifically, Wach *et al* (60) reported that patients with a baseline elevated MPV/PLT count ratio before cranial surgery had significantly shorter progression-free survival; however, they did not report any effect on OS. Differently, Alimohammadi *et al* (61) reported that elevated platelet distribution width (PDW)/PLT count ratio was an independent predictor of shortened OS, supporting the relationship between platelet activity and survival outcome in GBM. PDW reflects platelet size variations and is also a marker of platelet activation (63). A recent large retrospective analysis (64) concluded that no baseline blood tests could be reliably used as prognostic indicators in GBM. However, although the previous study was notable for the quality and large sample size, it should be noted that candidates for both biopsy and surgical resection, IDH mutated and wildtype tumors, were included. Furthermore only PLT count, aPTT and PT values were considered and MPV was not. Therefore, the heterogeneity in patient selection could have masked the prognostic role of PLT counts and coagulation markers on relevant oncological outcomes. The present study however, showed a relationship between MPV and OS in a homogeneous population of patients with IDH-wt GBM lesions undergoing craniotomy for maximal safe resection, which may represent a smaller, yet neuro-surgically relevant subgroup of patients with GBM, than those considered by Maas *et al* (64).

In accordance with the report of Wach *et al* (60), who reported increased MPV and lower PLT count in GBM models as the result of excessive systemic platelet activation and consumption, the present study found an inverse correlation between MPV values and PLT count in the patient cohort. A positive correlation of MPV values with increasing age and the overall tumoral volume was also observed. To investigate the role of aging in this physio-pathological mechanism, a subgroup analysis of patients aged >65 years was performed which confirmed that in this subpopulation the MPV value showed an even stronger predictive role of decreased survival in these patients. This latter phenomenon could be explained by a relatively different biology of GBM in elderly patients (65), as reported by Bozdag *et al* (66), who reported age-specific increased hypermethylation in polycomb group protein target genes and the upregulation of angiogenesis-related genes which could be associated with stronger systemic platelet activation in this subgroup of patients. At the same time, the contribution of the PLT count, PT, and aPTT were smaller than that which was observed in the younger population. These older patients did not differ from the young cohort for other baseline characteristics besides a worse performance status, which did not directly correlate with the MPV values in the analysis performed.

The positive correlation between tumor volumes and MPV values supports further future evaluation, as the extent of platelet activation could be associated with the lesion burden and relative aggressiveness.

The multivariate Cox regression model was used to help differentiate the single contributions to the overall OS length loss and indicated that increasing age, increasing MPV and deranged PLT count are independent predictors of worse OS in the patient cohort of the present study. However, the KPS, gender, and MGMT mutation status did not indicate a significant prognostic role in this cohort. Although the KPS is a well-known prognostic factor in GBM (67), in the present study only patients eligible for major surgery were included, ruling out those in extremely poor general conditions; therefore, the impact of KPS on OS could have been partially mitigated by patient selection. The MGMT methylation status was not determined in all included patients, of those for whom this was assessed, 37% presented MGMT methylation. This could suggest that the higher relative incidence of MGMT-unmethylated lesions could have reduced the survival benefit that would be expected from the surgery and adjuvant treatments in this patient cohort. Similarly, the female gender has been reported to be associated with better overall survival (68). In the present study, however, this relationship was only found in the subgroup of female patients with MGMT promoter methylation. These results indicate the importance of further investigations; however, this does not obscure the relevance of the role of MPV and PLT count in OS in patients with GBM. Overall, the results of the present study support the idea that systemic hemostasis and platelet activation might contribute to tumor aggressiveness, as indicated by the significant association between tumoral volumes, MPV, PLT count, and survival. To the best of our knowledge, no previous studies have reported an association between hemostasis markers and prognosis in a homogeneous population for both treatment and histology. Additional studies are required to elucidate the interplay between tumoral activity and platelet activation.

The principal limitations of the present study are the monocentric, retrospective design, the relatively small number of included patients, and the lack of thorough molecular analysis, such as full MGMT methylation profile and, TERT and EGFR mutation assessment. Data were retrospectively retrieved from the medical records of included patients and no control group was available. Additionally, this study lacks cross-sectional time research.

Results from this study suggest that, despite the intrinsic inter-individual and time-dependent variability of blood markers, PLT count and MPV could reveal the role of systemic hemostasis and platelet activation in promoting a pro-tumoral microenvironment in patients with GBM. These findings require additional studies to further validate this hypothesis and specifically characterize for GBM the pathological features of aggressiveness related to hemostasis activation, neo-angiogenesis, and the tumor immune microenvironment, and their impact on response to therapies and OS.

## Supplementary Material

Supporting Data

## Figures and Tables

**Table I.** Baseline characteristics of included patients, and lesions characteristics.

Epidemiology characteristics	Value	n	Percentage (%)
Age (years)			.
Mean	63±10.5		
<65		92	55
≥65		75	45
Gender			
Male		111	66
Female		56	34
Median KPS	80 (70–90)		
Tumor characteristics			
Mean FLAIR volume, cm^3^	98.3±59.2		
Mean T1-CE volume, cm^3^	34.5±26.7		
Satellite FLAIR lesions		28	17
Genetics (sample size)			
MGMT met (134)		49	37
Mean Ki67 (136)			31±18
Mean p53 (138)			20±24
Extent of resection (n=167)			
Complete		69	41
Near total		51	31
Partial		13	8
Subtotal		34	20
Postoperative protocol (n=149)			
Completed concurrent RT/TMZ		115	77
RT only/incomplete RT/TMZ		34	23
Adjuvant treatments (n=129)			
Adjuvant TMZ		107	83
6 cycles		54	42
12 cycles		9	7
Mean preoperative blood test markers			
PLT, ×10^9^/l	240±78.7		
MPV, ×10^−15^ l	10.3±0.98		
PT ratio	1.01±0.11		
aPTT ratio	0.94±0.10		
Overall survival			
Dead		129	77
Alive		38	23

Values are expressed as numbers and percentages (%). Continuous variables are shown as median (interquartile range) or mean ± standard deviation, according to the normality of distribution. MGMT met: MGMT methylation status. PLT, platelet counts; MPV, mean platelet volume; RT, radiotherapy; TMZ, temozolomide.

**Table II.** Univariate analysis of baseline characteristics of patients and lesions in low-MPV compared with high-MPV cohorts.

Preoperative characteristics	MPV ≤10.3 (×10^−15^ l) (n=77)	MPV >10.3 (×10^−15^ l) (n=88)	P-value
Age, mean ± SD	60.8±9.96	64.7±10.8	0.01
Male, n (%)	54 (70.1)	55 (62.5)	0.30
Female, n (%)	23 (29.9)	33 (37.5)	0.30
Performance status			
KPS, median (IQR)	80 (70–90)	80 (70–90)	0.45
Inflammation markers			
WBC, 10^9^/l	10.3±3.88	10.2±3.7	0.76
Neutrophils, 10^9^/l	7.98±4.01	8.20±3.97	0.73
Pathology			
Presence of central necrosis (n, %)	68 (90.6)	73 (85.8)	0.35
Midline shift > 5 mm	27 (36.4)	39 (45.3)	0.25
Mean FLAIR volume (cm^3^)	92.3±54.2	105.2±63.2	0.17
Mean T1-CE volume (cm^3^)	31.6±25.9	37.6±27.4	0.07
Presence of satellite FLAIR lesions (n, %)	16 (21.6)	11 (12.9)	0.14
Genetics			
MGMT methylation, (n, %)	23 (35.9)	26 (37.6)	0.85
p53 (% immunoreactive cells)	0.22±0.26	0.18±0.23	0.38
Ki67 (% immunoreactive cells)	0.31±0.18	0.31±0.19	0.92
Mean daily dose pre-operative dexamethasone (mg)	5.32±7.7	6.12±8.1	0.58

WBC, white blood cell count; KPS, Karnofsky performance status; MGMT met, MGMT methylation status; SD, standard deviation; IQR, interquartile range.

**Table III.** Multivariate Cox regression for MPV and therapies following surgery.

		Multivariate		
			
Variable	Reference	HR	95% CI	P-value
MPV (10^−15^ l)	NA	1.49	1.21-1.83	<0.001
PLT central range (165–300 10^9^/l)	Out of range	0.35	0.21-0.58	<0.001
Completed concurrent RT/TMZ	No/interrupted TMZ	0.37	0.22-0.60	0.001
Adjuvant TMZ	No additional TMZ	0.48	0.29-0.78	<0.001

PLT, platelet; MPV, mean platelet volume; RT, radiotherapy; TMZ, temozolomide; HR, hazard ratio.

**Table IV.** Multivariate Cox regression analysis shows an independent prognostic role of age, MPV, and PLT after controlling for other demographic and lesion parameters, and adjuvant therapies.

Variable	Reference	HR	95% CI	P-value
Age	NA	1.03	1.01-1.06	0.01
Female	Male	0.70	0.38-1.26	0.24
KPS <80	≥80	1.21	0.72-2.03	0.45
Pathology				
MGMT met	Non met	0.57	0.31-1.05	0.07
Ki67 (%)	NA	3.77	0.83-17.1	0.08
p53 (%)	NA	0.83	0.30-2.30	0.73
Markers				
MPV (10^−15^ l)	NA	1.56	1.13-2.16	0.006
PT ratio	NA	6.02	0.70-51.1	0.10
aPTT ratio	NA	3.24	0.11-89.6	0.48
PLT central range (165–300 10^9^/l)	Out of range	0.26	0.13-0.54	<0.001
Radiology				
FLAIR volume (cm^3^)	NA	1.00	0.99-1.01	0.74
T1-CE volume (cm^3^)	NA	1.01	0.99-1.02	0.08
Satellite FLAIR lesions	No satellite lesions	1.23	0.58-2.61	0.57
Complete resection	Near total/subtotal/partial	0.52	0.30-0.90	0.01
Completed concurrent RT/TMZ	No/interrupted TMZ	0.24	0.13-0.45	<0.001

KPS, Karnofsky performance status; MGMT met, MGMT methylation status; PLT, platelet; MPV, mean platelet volume; aPTT, activated partial thromboplastin time; FLAIR, fluid-attenuated inversion recovery; RT, radiotherapy; TMZ, temozolomide; HR, hazard ratio.

## Data Availability

The data generated in the present study may be requested from the corresponding author.

## Authors' information

Dr Pierfrancesco De Domenico - ORCID: 0000-0003-3330-162X.
