# Supplementary material for: Pre‑operative mean platelet volume is associated with overall survival in patients with IDH‑wildtype glioblastoma undergoing maximal safe resection
Source: Oncol Lett. 2024 Sep 30;28(6):576. doi: 10.3892/ol.2024.14709 (PMC11467840; doi:10.3892/ol.2024.14709)

Figure S1. Kaplan-Meier curves for overall survival analysis. (A) Age, (B) Karnofsky performance status, (C) gender. (D) MGMT methylation status (E) gender in MGMT methylated sub-population and (F) unifocal lesion vs. presence of additional satellite FLAIR lesions. P-values represent log-rank test results. *P-value for the two-stage procedure performed when the proportional hazard assumption was violated. HR, hazard ratio with 95% CI interval; FLAIR, fluid-attenuated inversion recovery.

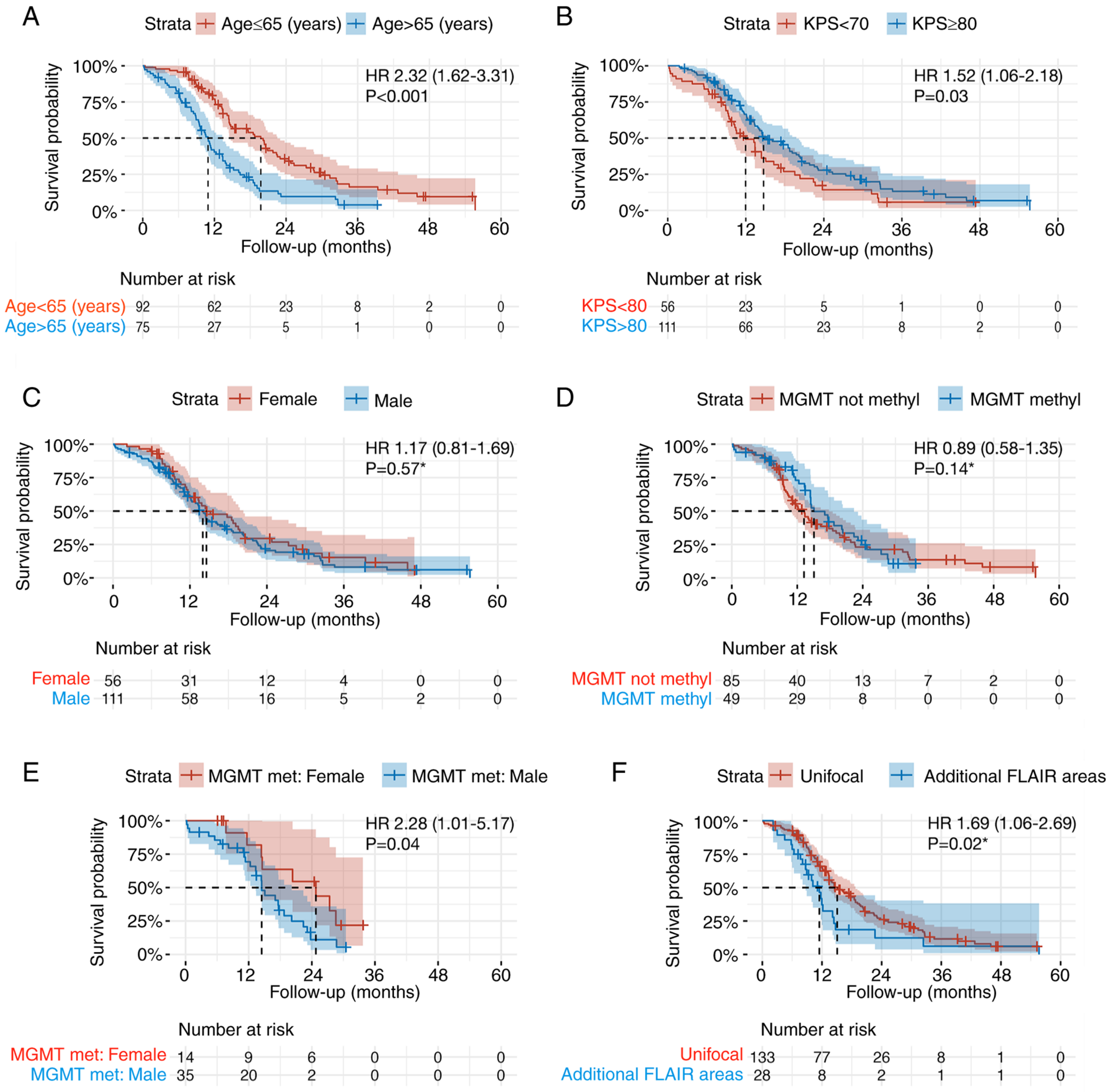

Figure S2. Kaplan-Meier curves for overall survival analysis in the older patient population (>65 years). (A) PLT count normal range 165-300x10^9/l vs. out-of-range. (B) MPV high vs. low. (C) PT-ratio high vs. low. (D) aPTT-ratio high vs. low. P-values represent log-rank test results. *P-value for the two-stage procedure performed when the proportional hazard assumption was violated. HR, hazard ratio with 95% CI interval; PLT, platelet; MPV, mean platelet volume; aPTT, activated partial thromboplastin time.

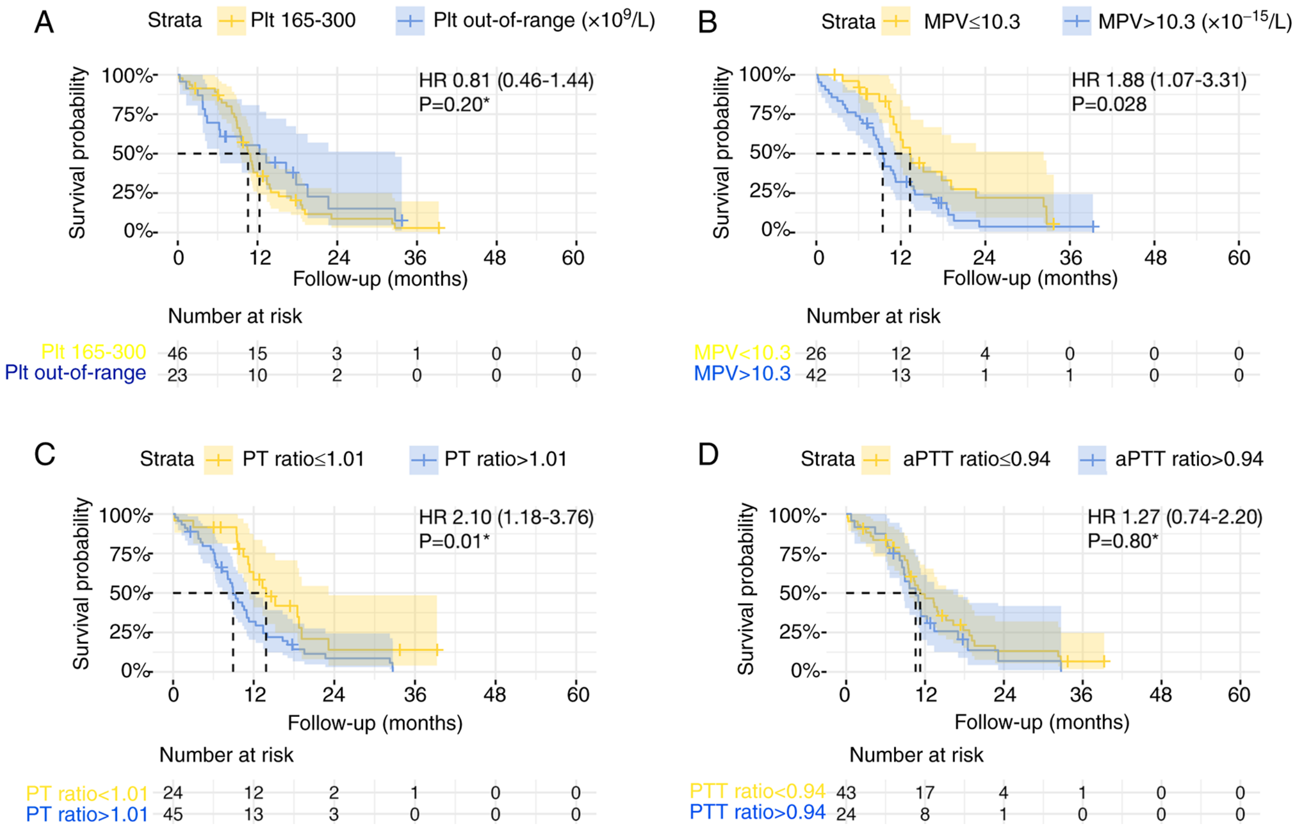

Supplement: Supporting Data [file Supplementary_Data.pdf]
